# Expanding generalized contrast-to-noise ratio into a clinically relevant measure of lesion detectability by considering size and spatial resolution

**DOI:** 10.1117/1.JMI.11.5.057001

**Published:** 2024-10-23

**Authors:** Siegfried Schlunk, Brett Byram

**Affiliations:** Vanderbilt University, Nashville, Tennessee, United States

**Keywords:** lesion detectability, signal-to-noise ratio, generalized contrast-to-noise ratio, ultrasound imaging, image quality, spatial resolution

## Abstract

**Purpose:**

Early image quality metrics were often designed with clinicians in mind, and ideal metrics would correlate with the subjective opinion of practitioners. Over time, adaptive beamformers and other post-processing methods have become more common, and these newer methods often violate assumptions of earlier image quality metrics, invalidating the meaning of those metrics. The result is that beamformers may “manipulate” metrics without producing more clinical information.

**Approach:**

In this work, Smith et al.’s signal-to-noise ratio (SNR) metric for lesion detectability is considered, and a more robust version, here called generalized SNR (gSNR), is proposed that uses generalized contrast-to-noise ratio (gCNR) as a core. It is analytically shown that for Rayleigh distributed data, gCNR is a function of Smith et al.’s $C_{\psi }$ (and therefore can be used as a substitution). More robust methods for estimating the resolution cell size are considered. Simulated lesions are included to verify the equations and demonstrate behavior, and it is shown to apply equally well to *in vivo* data.

**Results:**

gSNR is shown to be equivalent to SNR for delay-and-sum (DAS) beamformed data, as intended. However, it is shown to be more robust against transformations and report lesion detectability more accurately for non-Rayleigh distributed data. In the simulation included, the SNR of DAS was 4.4±0.8, and minimum variance (MV) was 6.4±1.9, but the gSNR of DAS was 4.5±0.9, and MV was 3.0±0.9, which agrees with the subjective assessment of the image. Likewise, the ${\mathrm{DAS}}^{2}$ transformation (which is clinically identical to DAS) had an incorrect SNR of 9.4±1.0 and a correct gSNR of 4.4±0.9. Similar results are shown *in vivo*.

**Conclusions:**

Using gCNR as a component to estimate gSNR creates a robust measure of lesion detectability. Like SNR, gSNR can be compared with the Rose criterion and may better correlate with clinical assessments of image quality for modern beamformers.

## 1 Introduction

The clinical value of a medical ultrasound image is determined by the clinician viewing the image and depends on the clinical task being performed and their ability to make a correct assessment from the available image. However, in research and development, it is not feasible to have a clinician or team of clinicians assess every image. Instead, image quality metrics that correlate with clinician assessment are more efficient for large datasets or when quick iteration is needed. A common line of thinking is that clinical image quality should correlate with the ability to detect simple lesions. That is, a beamformer that reports more detectable lesions likely produces higher-quality images. This is why image quality metrics such as contrast-to-noise ratio (CNR) are often used. Though, in practice, image quality is a complex interaction between many aspects of the image and imaging system, this work focuses on the challenge of simple lesion detection and aims to create a robust metric for that task.

Smith and Lopez^1^ recognized that lesion detection relies not only on the contrast but also on the size of the lesion, and so, they proposed the contrast-detail phantom for grading ultrasound systems. This phantom uses cones of varying contrasts, and clinicians decide what combinations of lesion size and contrast they can detect. Using the information from this phantom, Smith et al.^2^ proposed a metric that correlated with clinician assessment of detectability. Derived from the statistics of ultrasound speckle, Smith et al. referred to this metric as signal-to-noise ratio (SNR) and showed that it can even be used with the Rose criterion,^3^ a common threshold for detectability used in general imaging applications. Though the abbreviation SNR has been used to mean many different things, in this work, SNR refers only to Smith et al.’s metric. A thorough description of SNR will follow in Sec. 2.1.

A critical drawback of SNR is that it is explicitly designed for delay-and-sum (DAS) beamforming,^4^ where the data are known to have certain statistical properties. Because of these properties, many image quality metrics tend to correlate well with the underlying information of the DAS image. However, when using other adaptive beamformers, the resulting images may no longer possess known speckle statistics, and traditional image quality metrics may no longer accurately reflect the information of the image. In fact, it is surprisingly easy to create transformations that improve SNR (or other traditional metrics) that do not change the underlying information (i.e., clinical value).^2^^,^^5^^,^^6^ The use of ineffective metrics on adaptive beamformers is recognized as an issue by many groups and has led to the development of more robust image quality metrics that can more accurately compare these advanced beamformers. For example, the dynamic range test,^7^^,^^8^ contrast ratio (CR) dynamic range,^9^^,^^10^ histogram matching,^11^ and generalized contrast-to-noise ratio (gCNR)^5^ were all proposed to try and address this or similar concerns. However, while more robust to the information content of an image, many of these have not been tested with respect to clinician assessment, making it difficult at times to interpret the clinical meaning of these measurements.

The goal of this work is to create a generalized version of SNR (gSNR) that can be applied universally to modern adaptive beamformers. For the first time, we will analytically show that in Rayleigh distributed environments, gCNR is a function of CR, which in turn is a function of the contrast component used to calculate SNR. This allows gCNR to be used as a robust substitution to calculate gSNR, which will be derived in the following pages. In addition, more robust options for calculating lesion size and system resolution will be considered. This novel generalization of SNR is robust to transformations (like gCNR) and will retain its relationship to clinical assessments via the contrast-detail phantom and Rose criterion (like SNR). The challenges involved with finding robust equivalents for the components of SNR will be explored, and solutions will be proposed for each. An implementation of gSNR will be proposed, and both simulations and *in vivo* data involving multiple non-DAS beamformers will be provided to demonstrate its behavior.

## 2 Image Quality Metrics

### 2.1 Signal-to-Noise Ratio

Smith et al.’s SNR for lesion detectability^2^ can be written as

$$\mathrm{SNR}=C_{\psi }M^{1/2}N^{1/2}.$$ (1)

Smith et al. considered $C_{\psi }$ to be a signal-to-noise ratio component, whereas M is the number of speckle correlation cells in the lesion area and N is the number of independently compounded images (and will simply be 1 in this work). $C_{\psi }$ is based on the average intensity I of the lesion (L) and background (B)

$$C_{\psi }=\frac{I_{L}-I_{B}}{{\left(I_{L}^{2}+I_{B}^{2}\right)}^{1/2}}.$$ (2)

This bounds $C_{\psi }$ between 0 and 1 (0 for no contrast difference, and 1 for a truly anechoic lesion).^2^

$M=S/S_{c}$, the number of speckle correlation cells, is calculated from the size of the lesion, S, and the correlation cell size, $S_{c}$. They found that the correlation cell size is based on the autocovariance of the speckle and determined that it is also comparable to the resolution cell size, which they confirmed in phantoms.^12^ Therefore, they assumed that the speckle cell size is functionally an ellipse with radii equal to half the lateral and axial resolution of the system. In their work, they measured resolution as the full width at half maximum (FWHM) of the point spread function (PSF) and pulse length.

A particularly useful aspect of SNR is that it can be used to grade an image without the need for another reference image. Most metrics primarily have meaning when comparing multiple images against each other, but as SNR relates to the contrast-detail phantom, the SNR value itself has meaning and can infer the clinical quality of the image. In fact, it can even be linked back to the well-known Rose criterion. The Rose criterion comes from a statistical analysis where Albert Rose estimated that a signal amplitude of four to five times the root mean square of the noise was required to identify the signal with near certainty.^3^ To guard against false positives, Rose suggested that the threshold of five is more reliable, especially for larger pictures. Smith et al.^2^ showed that a factor of $\sqrt{2}$ converts to the SNR used in this work, giving a converted threshold of ${\mathrm{SNR}}_{T}=5/\sqrt{2}\approx 3.54$ for the SNR defined here.

### 2.2 Generalized Contrast-to-Noise Ratio

gCNR^5^ is described as a lesion detectability metric and a more robust version of the traditional CNR, which itself is popular due to the expectation that CNR correlates with subjective image quality.^13^ gCNR is measured as gCNR=1−OVL, where OVL is the overlap of the probability density functions (PDFs) of the lesion and background regions. gCNR values approaching 1 have overlap approaching 0, meaning the PDFs are completely separated. As the relative distribution of these two PDFs is unchanged when the image undergoes a transformation, the gCNR cannot be manipulated in the way that other metrics can, making it more robust. Though finding the true PDFs of the two regions can be difficult, we have previously proposed robust methods for estimating the gCNR even in extreme scenarios.^14^ (These methods are publicly available from our GitHub repository https://github.com/VU-BEAM-Lab/gCNR_solver. Here, we will use the “ecdf” implementation.)

As with SNR, gCNR can be used without a reference image. As gCNR is a measure of the overlap of the two regions of data, it not only provides a measure of the likelihood of separating a data point into one region or the other, but it can also be applied to any kind of data. However, Smith et al.’s work demonstrates that clinical interpretation of detectability also depends on lesion size and system resolution, which does not impact gCNR. This was observed in our prior work, where as long as there are enough data sampled to accurately represent the population, gCNR will converge on a fixed value.^14^ This means that gCNR and SNR must be measuring different aspects of the image.

### 2.3 Generalized Signal-to-Noise Ratio

Smith et al. derived SNR with the statistics of speckle in mind: that image data are beamformed with DAS and the envelope is Rayleigh distributed. However, in theory, it might be possible to derive a version of SNR for other beamformers and therefore other distributions (Exponential, Gaussian, etc.); in practice, it is unreasonable (and perhaps impossible) to re-derive the metric for every possible situation. However, it is also understandable that SNR cannot simply be applied to other beamforming methods either as these methods can manipulate both $C_{\psi }$ and the correlation cell size without changing the underlying information. gCNR is robust to these manipulations but lacks the explicit clinical meaning of SNR. As a result, our goal is to merge the clinical meaning of SNR and the robustness of gCNR into a single metric we will call gSNR.

In Eq. (1), for SNR, $C_{\psi }$ is a contrast measurement ranging from 0 to 1 calculated on the intensity data of an image.^2^ gCNR can also be considered a quantitative measure of contrast ranging from 0 to 1 and can be equally calculated on magnitude or intensity data (as intensity is just a transformation of magnitude).^5^ Then, we define gSNR as

$$\mathrm{gSNR}=f(\mathrm{gCNR}){\overset{˜}{M}}^{1/2}N^{1/2},$$ (3)

where f is a function $f(\mathrm{gCNR})=C_{\psi }$ for the case of DAS data. Assuming such a function can be found, this would allow us to estimate a gSNR value that is robust to transformations. However, also note that we differentiate $\overset{˜}{M}$ from the original to indicate the use of a more robust calculation to estimate the value. These two components will be discussed in more detail in Secs. 2.3.1 and 2.3.2.

#### 2.3.1 Analytic calculation of F

The well-understood nature of the DAS distribution makes it possible to calculate its behavior analytically. Enveloped DAS data follow the Rayleigh distribution,^12^ which implies that the intensity, the square of the enveloped data, follows the exponential distribution. Table 1 includes several definitions from these distributions that will be used in this section. As these distributions are related, this allows us to define them in relation to each other. Consider a lesion (L) and background region (B) that are described by Rayleigh distributions with scale parameters $\sigma_{L}$ and $\sigma_{B}$, respectively. Then, the corresponding intensity data are governed by exponential distributions with rate parameters $\lambda_{L}=1/(2\sigma_{L}^{2})$ and $\lambda_{B}=1/(2\sigma_{B}^{2})$, which come directly from the definition of the distributions. Furthermore, the mean value (expected value) of an exponential distribution is simply I=E[X]=1/λ, which means that Eq. (2) for $C_{\psi }$ from earlier becomes

$$C_{\psi }=\frac{I_{L}-I_{B}}{(I_{L}^{2}+I_{B}^{2}{)}^{1/2}}=\frac{2\sigma_{L}^{2}-2\sigma_{B}^{2}}{((2\sigma_{L}^{2}{)}^{2}+(2\sigma_{B}^{2}{)}^{2}{)}^{1/2}}=\frac{\sigma_{L}^{2}-\sigma_{B}^{2}}{(\sigma_{L}^{4}+\sigma_{B}^{4}{)}^{1/2}}.$$ (4)

**Table 1:** Definitions for distributions.

	R∼ Rayleigh (σ)	X∼ Exponential (λ)
Mean (μ)	$\sigma \sqrt{\pi /2}$	1/λ
PDF	$\left(x/\sigma^{2})\exp(-x^{2}/(2\sigma^{2})\right)$	λ exp(−λx)
CDF	$1-\exp(-x^{2}/(2\sigma^{2}))$	1−exp(−λx)

Often, CR is defined as the log-compressed ratio of the mean values of two regions, but functionally, it is simply the ratio of the means. For Rayleigh distributions, the ratio of the means is simply the ratio of the scale parameters, and we can define $\mathrm{CR}=\mu_{L}/\mu_{B}=(\sigma_{L}\sqrt{\pi /2})/(\sigma_{B}\sqrt{\pi /2})=\sigma_{L}/\sigma_{B}$. As $\sigma_{L},\sigma_{B},C_{\psi }$ are all positive, real values [Eq. (4)] can be written as

$${\mathrm{CR}}^{2}=\frac{\sigma_{L}^{2}}{\sigma_{B}^{2}}=\frac{\sqrt{2C_{\psi }^{2}-C_{\psi }^{4}}-1}{C_{\psi }^{2}-1},$$ (5)

showing that we can relate CR and $C_{\psi }$.

It is possible to find a similar equation for gCNR using the same Rayleigh distribution scale parameters. A critical step to do this comes from the proof in our prior work and can be found in its supplementary material.^14^ In theorem 1.4 of our prior work, we showed that gCNR can be expressed as

$$\mathrm{gCNR}=\sum H_{\mathrm{MAX}}-\sum H_{\mathrm{MIN}},$$ (6)

where $H_{\mathrm{MAX}}$ and $H_{\mathrm{MIN}}$ are the local maximums and minimums of the function H=F−G, respectively. F and G are the cumulative distribution functions (CDFs) for any arbitrary sets of data that gCNR is being calculated on. To simplify the proof, we previously defined the data as scaled between 0 and 1, which we maintain here. In this case, we have Rayleigh distributions defined by $\sigma_{L}$ and $\sigma_{B}$, and the equation for the CDF for a Rayleigh distribution can be substituted in to find

$$H=CDF_{L}-CDF_{B}=(1-e^{\left(-x^{2}/(2\sigma_{L}^{2}\right)})-(1-e^{\left(-x^{2}/(2\sigma_{B}^{2}\right)})=e^{\left(-x^{2}/(2\sigma_{B}^{2}\right)}-e^{\left(-x^{2}/(2\sigma_{L}^{2}\right)}.$$ (7)

Lemma 1.1 from our previous proof tells us that the local extrema of H are located at the intersections of the corresponding PDFs. The original proof for Eq. (6) is applied to any arbitrary distribution, but in the case of Rayleigh distributions, there are exactly three locations where the PDFs intersect (assuming the distributions are not identical): $x=\{0,x_{0},1\}$. For any PDF, PDF(0)=0 and PDF(1)=0 are clear, meaning that any two PDFs will intersect at those locations. As all Rayleigh distributions are defined by a single scale parameter, that leaves exactly one other intersection $x_{0}\in (0,1)$. Then, this intersection can simply be solved using the equations of the PDFs for Rayleigh distributions ${\mathrm{PDF}}_{L}(x_{0})={\mathrm{PDF}}_{B}(x_{0})$

$$\frac{x_{0}}{\sigma_{L}^{2}}e^{-x_{0}^{2}/(2\sigma_{L}^{2})}=\frac{x_{0}}{\sigma_{B}^{2}}e^{-x_{0}^{2}/(2\sigma_{B}^{2})},$$ (8)

which can be solved for $x_{0}$ as

$$x_{0}^{2}=\frac{4\sigma_{L}^{2}\sigma_{B}^{2}\;\ln(\sigma_{B}^{2}/\sigma_{L}^{2})}{2\sigma_{B}^{2}-2\sigma_{L}^{2}}.$$ (9)

As $CDF_{L}(0)=CDF_{B}(0)=0$ and $CDF_{L}(1)=CDF_{B}(1)=1$ by definition, H(0)=H(1)=0. Then, from the list of potential intersections of the PDFs, $x=\{0,x_{0},1\}$, we only have one location that provides a non-zero value of H: $x=x_{0}$. So, Eq. (6) simplifies as we only need to calculate the value of H at $x_{0}$

$$\mathrm{gCNR}=\sum H_{\mathrm{MAX}}-\sum H_{\mathrm{MIN}}=[H(x_{0})]-[H(0)+H(1)]=H(x_{0})=e^{\left(-x_{0}^{2}/(2\sigma_{B}^{2}\right)}-e^{\left(-x_{0}^{2}/(2\sigma_{L}^{2}\right)},$$ (10)

which follows from Eq. (7). Then, we can substitute Eq. (9) where we solved for $x_{0}^{2}$ into this equation to get a function of gCNR in terms of the two Rayleigh parameters $\sigma_{L}$ and $\sigma_{B}$

$$\mathrm{gCNR}=e^{\left(-(\frac{4\sigma_{L}^{2}\sigma_{B}^{2}\;\ln(\sigma_{B}^{2}/\sigma_{L}^{2})}{2\sigma_{B}^{2}-2\sigma_{L}^{2}}))/(2\sigma_{B}^{2}\right)}-e^{\left(-(\frac{4\sigma_{L}^{2}\sigma_{B}^{2}\ln(\sigma_{B}^{2}/\sigma_{L}^{2})}{2\sigma_{B}^{2}-2\sigma_{L}^{2}}))/(2\sigma_{L}^{2}\right)},$$ (11)

which can be simplified into

$$\mathrm{gCNR}=\frac{\sigma_{B}^{2}/\sigma_{L}^{2}-1}{(\sigma_{L}^{2}/\sigma_{B}^{2}{)}^{1/((\sigma_{L}^{2}/\sigma_{B}^{2})-1)}}=\frac{1/{\mathrm{CR}}^{2}-1}{({\mathrm{CR}}^{2}{)}^{1/({\mathrm{CR}}^{2}-1)}}.$$ (12)

As Eq. (5) for $C_{\psi }$ and Eq. (12) for gCNR are both written in terms of ${\mathrm{CR}}^{2}=\sigma_{L}^{2}/\sigma_{B}^{2}$, one final substitution gives

$$\mathrm{gCNR}=\frac{\frac{C_{\psi }^{2}-1}{\sqrt{2C_{\psi }^{2}-C_{\psi }^{4}}-1}-1}{{\left(\frac{\sqrt{2C_{\psi }^{2}-C_{\psi }^{4}}-1}{C_{\psi }^{2}-1}\right)}^{1/(\frac{\sqrt{2C_{\psi }^{2}-C_{\psi }^{4}}-1}{C_{\psi }^{2}-1}-1)}}.$$ (13)

**Fig. 1:**
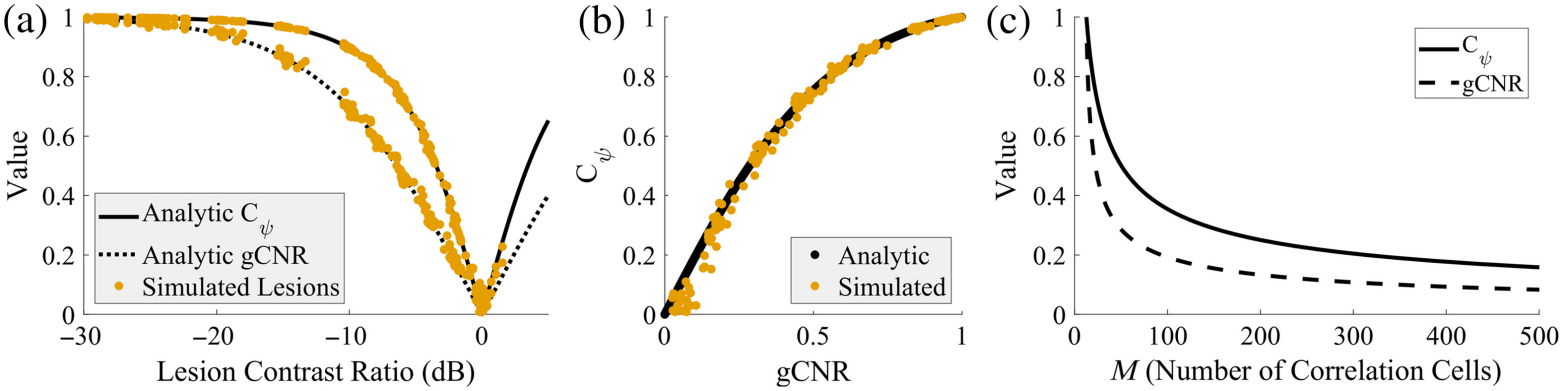
Comparing the analytic relationship to simulated lesion results for converting among CR, $C_{\psi }$, and gCNR using Eqs. (5) and (12) (a) and Eq. (13) (b). The simulated lesions include varying radii (2 to 5 mm) across a range of CRs, showing good agreement between the analytic equations and the simulated results. Note that simulations were performed at amplitude increments of 5 dB, which produces the gaps in the figure. (c) Using Eq. (1) and the Rose criterion threshold (3.54) to predict the threshold values of $C_{\psi }$ and gCNR. For a given value of M (the number of correlation or resolution cells), having a measured $C_{\psi }$ or gCNR above the corresponding line indicates that the lesion is above the Rose criterion.

This proves that for Rayleigh distributions, gCNR, $C_{\psi }$, and CR all provide equivalent information. In addition, we have an exact form for $f^{-1}$, and we will later provide an empirical approximation for f that is much more reasonable to use in practice. Figure 1 shows a quick example of applying the three analytic equations to some simple simulated lesions to show agreement with more practical data. It also shows some conclusions that can be drawn from these equations to predict the minimum required $C_{\psi }$ or gCNR, based on the size (number of correlation cells) of the lesion.

#### 2.3.2 Robust estimation of $\overset{˜}{M}$

Table 2 is a quick reference to all notations and abbreviations described in this section. The notations with the tilde (e.g., $\overset{˜}{M}$) indicate the robust variations that can be used for gSNR.

**Table 2:** Reference for notation and abbreviation for size and resolution methods.

Number of correlation cells and resolution methods		
M notation	Method abbreviation	Description
$M_{\mathrm{FWHM}}$	FWHM	FWHM (standard for DAS data)
${\overset{˜}{M}}_{\mathrm{FWHM},\text{matched}}$	${\mathrm{FWHM}}_{\mathrm{matched}}$	FWHM histogram matched to DAS
$M_{\mathrm{corr}}$	autocorr	Autocorrelation (standard for DAS data)
${\overset{˜}{M}}_{\text{Sparrow}}$	Sparrow	Sparrow’s criterion
${\overset{˜}{M}}_{\mathrm{info}}$	autoinfo	Autoinformation length
${\overset{˜}{M}}_{\text{Sparrow},\text{scaled}}$	${\text{Sparrow}}_{\text{scaled}}$	Sparrow’s criterion scaled to DAS FWHM
${\overset{˜}{M}}_{\text{info},\text{scaled}}$	${\text{autoinfo}}_{\text{scaled}}$	Autoinformation scaled to DAS FWHM

A major component of SNR is accurately assessing the correlation cell size, $S_{c}$, as the number of independent correlation cells in the lesion directly follows: $M=S/S_{c}$. The correlation cell size is derived from the autocovariance of the speckle (which is related to autocorrelation), but Wagner et al.^12^ demonstrated that the –6 dB resolution cell size is comparable to $S_{c}$, which can be easily measured from the FWHM of the PSF and pulse length. For DAS, this can be estimated from features of the imaging system as it is expected that the resolution is only dependent on the imaging system itself. In this work, M (for calculating SNR) is estimated from the FWHM in point target simulations ($M_{\mathrm{fwhm}}$) and from the autocorrelation of the speckle for *in vivo* cases ($M_{\mathrm{corr}}$).

However, the FWHM and autocorrelation function can be affected by transformations much like CNR and SNR. To compensate for this effect, histogram matching^11^ can be used to match other beamformers to DAS, functionally removing the effects of transformations. We found using the “full” histogram matching method that using DAS as the reference image did well to eliminate problematic transformations in most cases and produced similar lateral resolution measurements compared with the other robust methods. However, we found it unreliable to apply to some beamformers for measuring axial resolution, and it continues to be the case that determining the optimal method of matching is challenging. Sparrow’s criterion (or resolution limit) is a measure of resolution that is independent of transformations because it defines resolution as the distance between two points when a minimum becomes detectable between them.^15^ Specifically, we measure this distance as when a minimum becomes visible between two points positioned at the same depth. Finally, the autoinformation length is included as an information-based resolution metric, being similar but more robust compared with autocorrelation and easier to apply than Sparrow’s criterion.^16^ Histogram matching (${\overset{˜}{M}}_{\mathrm{fwhm},\text{matched}}$), Sparrow’s criterion (${\overset{˜}{M}}_{\text{sparrow}}$), and autoinformation length (${\overset{˜}{M}}_{\text{info}}$) are included to find the most robust option. In all cases, we then estimated $S_{c}\approx \pi \times ({\mathrm{res}}_{\mathrm{lat}}/2)\times ({\mathrm{res}}_{\mathrm{ax}}/2)$ from the measured lateral and axial resolution.

The original works^2^^,^^12^ used the FWHM and autocorrelation for DAS to estimate the resolution cell size, suggesting that these methods are correct for comparing against the Rose criterion^3^ and the original contrast-detail phantom.^1^ However, Sparrow’s criterion and the autoinformation length generally predict smaller resolution cells, even for DAS. As a result, we calculate a scalar for each that when applied matches the DAS resolution of each method to the “true” value. This same scalar is applied to each beamformer, and for completeness, calculations will be performed both without and with the scalar applied. Scaled measurements will be indicated by “scaled” where appropriate (e.g. ${\overset{˜}{M}}_{\text{info},\text{scaled}}$).

## 3 Evaluation Methods

MATLAB (The MathWorks, Natick, Massachusetts, United States) was used for all simulations and implementations.

### 3.1 Simulated Lesions and Point Targets

Field II^17^^,^^18^ was used for both lesion and point target simulations. In all cases, targets were simulated at a depth of 30 mm at the focus. A linear array transducer was simulated with 117 active elements with a pitch of 0.257 mm, transmitting at 3 MHz with a bandwidth of 60%.

Lesion simulations were generated with radii ranging from 1 to 5 mm with approximate amplitudes ranging from –30 to 0 dB, and n=6 independent realizations of speckles were generated for each case. In these simulations, images were produced by acquiring 128 beams, 0.234 mm apart. This gives an expected resolution cell size of $0.133\;{\mathrm{mm}}^{2}$, and scatterers were placed to achieve an average of 15 scatterers per cell. For all image quality metrics, the lesion region L and background B were selected as the area of the lesion and a region of background, respectively. The number of data points in region L therefore scales directly with the square of the radius of the lesion. The regions chosen are shown later in Fig. 3. The region of interest (ROI) for each of the 1, 2, 3, 4, and 5 mm radius lesions had areas of 2.92, 11.28, 25.76, 45.58, and $70.98\;{\mathrm{mm}}^{2}$. These are based on 80% of the true size of the simulated lesions as we wanted to minimize the amount of sidelobe clutter present in the ROI and will be further explained in Sec. 3.4. Background regions were chosen to include approximately the same number of data points as the corresponding lesion.

Point target simulations were split into two categories: single-point targets for use with measuring the FWHM of the PSF and double-point targets for use with measuring the resolution limit using Sparrow’s criterion. In both cases, 31 beams were simulated, and points were positioned at the focal depth. For the single target cases, the point was located along the middle beam, and the beams were positioned 0.0234 mm apart for higher accuracy measurements. For the double target cases, beams were positioned such that the points were located along the first and last beams, with 29 beams in between them, to make it easier to identify minimums in between the two points. For these cases, lateral points were generated with separations ranging from 0.381 to 0.440 mm with increments of 0.001 mm, and axial points had separations ranging from 0.290 to 0.310 mm with increments of 0.001 mm. This allowed us to get an estimate of the resolution limit on the order of 0.001 mm. To create additional realizations, n=6 independent white noise realizations were generated and added to the point targets at SNRs of 60 dB. As the white noise can create misleading minimums when measuring Sparrow’s criterion, these cases were fit using the MATLAB fit function (The MathWorks, Inc.) and the “poly4” fit type.

### 3.2 *In Vivo* Cases

To demonstrate that gSNR can equally be applied to *in vivo* data, we captured *in vivo* liver data using a Verasonics Vantage Ultrasound System (Verasonics, Inc., Kirkland, Washington, United States) with a C5-2 curvilinear transducer. A total of 128 angles uniformly spaced to span 75 deg were acquired with a center frequency of 4.1667 MHz, focused at 6 cm. Six cases were acquired from one patient for use in estimating the average spatial resolution, and one of those cases was chosen for analysis.

For resolution, a patch of homogeneous speckle was chosen near the focus depth, and both autocorrelation and the autoinformation length were used to estimate lateral and axial resolutions. Again, it is assumed that the autocorrelation of the DAS case produces the closest value to the truth,^2^^,^^12^ so a scalar is applied to the autoinformation lengths such that the DAS measurements are consistent between the two methods. For the analysis case, three blood vessels of varying sizes were chosen, and SNR using $M_{\text{corr}}$ and gSNR using ${\overset{˜}{M}}_{\text{info},\text{scaled}}$ were calculated.

### 3.3 Beamformers and Post-Processing Methods

We include brief descriptions of image formation methods included in the analysis here but leave the majority of the finer details to prior works for brevity. Throughout the rest of the work, these methods will be referred to as “beamformers” rather than the more correct “beamformers and post-processors” to improve flow and readability.

#### 3.3.1 Delay-and-Sum

DAS was implemented without apodization.^4^ DAS also serves as the reference (ref) for all histogram-matching applications.

#### 3.3.2 Generalized coherence factor

The generalized coherence factor (GCF) is a weighting of the DAS image designed to reduce focusing errors caused by phase aberrations.^19^ A cutoff of $M_{0}=5$ was chosen as a reasonable value for general imaging cases as we and others have used in prior works.^10^^,^^14^^,^^20^^,^^21^

#### 3.3.3 Minimum variance

Minimum variance (MV) is a method for improving lateral resolution and was implemented using the adaptations for ultrasound imaging.^22^^,^^23^ Subarray lengths, N, of 1/2 the aperture length were used, and diagonal loading of ϵ=Δ·tr(R^), where Δ=1/(10N), was applied. $\mathrm{tr}(\hat{R})$ is the trace of the matrix.

#### 3.3.4 Filtered delay multiply and sum

Filtered delay multiply and sum (F-DMAS) is an adaptive beamformer that combinatorially couples and multiplies the delayed channel data before summing across the channels.^24^ The direct current (DC) and high-frequency components are removed using a band-pass filter centered around $2f_{c}$, where $f_{c}$ is the center frequency.

#### 3.3.5 Simple gray level transformations

We and other groups have previously used gray level transformations to demonstrate how dynamic range transformations can manipulate many traditional image quality metrics.^5^^,^^14^^,^^16^ Specifically, we will define a simple transform on the enveloped DAS data, $\left|S_{\mathrm{DAS}}\right|$

$${\mathrm{DAS}}^{n}(|S_{\mathrm{DAS}}|)=|S_{\mathrm{DAS}}{|}^{n},$$ (14)

where n is any desired power. Here, the square root and square cases are considered, i.e., $\sqrt{\mathrm{DAS}}$ and ${\mathrm{DAS}}^{2}$. These transformations result in simple compression or stretching of the dynamic range, but as stated earlier, these kinds of transformations do not alter the clinical value or the information content of the images. As with the other methods included here, the resulting value is log compressed with $20*\log_{10}$ when displayed.

### 3.4 Choosing an Optimal ROI

**Fig. 2:**
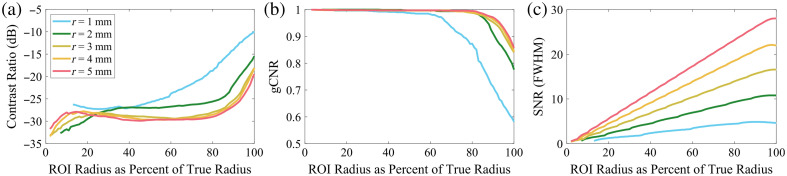
Example of how choosing the radius of the ROI for an anechoic lesion impacts resulting (a) CR, (b) gCNR, and (c) SNR. Lesions of varying radii (1 to 5 mm) are shown, chosen ROI radius is displayed as a percent of the true radius. Both CR and gCNR are affected by off-axis clutter, which results in decreased performance as the ROI radius approaches the true radius of the lesion. However, SNR is related to the size of the ROI and so continues to increase as the ROI radius increases. An ROI radius of 80% is chosen for later analysis as it produces the best balance between optimizing both contrast-like and size-dependent metrics. The curve for the 1 mm lesions shows how off-axis clutter can complicate analysis in smaller ROIs.

When considering image quality metrics such as CNR or gCNR, the choice of the ROI for the target and reference regions is critical, though often inadequately discussed. In simulations where the true size of targets is known, it is possible to create an ROI that is exactly the size of the target. However, off-axis clutter effects (and potentially others) mean that for some metrics, using the true size for the ROI produces poorer metrics than if a slightly smaller ROI was used. Off-axis clutter generally shrinks the apparent size of the lesion, worsens the average amplitude of the lesion, and increases the variance within the true lesion ROI. Image quality metrics that depend on amplitude and variance therefore are encouraged to choose a smaller radius compared with the true radius to reduce the impact that these off-axis effects have. Consider Fig. 2, CR, gCNR, and SNR are shown as a function of the percent of the ROI radius compared with the true radius for anechoic lesions of varying radii. CR and gCNR depend mostly on amplitude and variance, so there is a distinct moment where the ROI radius begins to include off-axis clutter and the performance drops. In comparison, SNR depends on the size and therefore mostly continues to improve as the ROI radius increases. As both gCNR and SNR are being considered here, we will choose to use a universal 80% ROI radius compared with the true radius as it serves to optimize both metrics simultaneously.

## 4 Results

### 4.1 Verifying the Analytic Solution *gCNR* = *f*^−1^(*C_ψ_*)

**Fig. 3:**
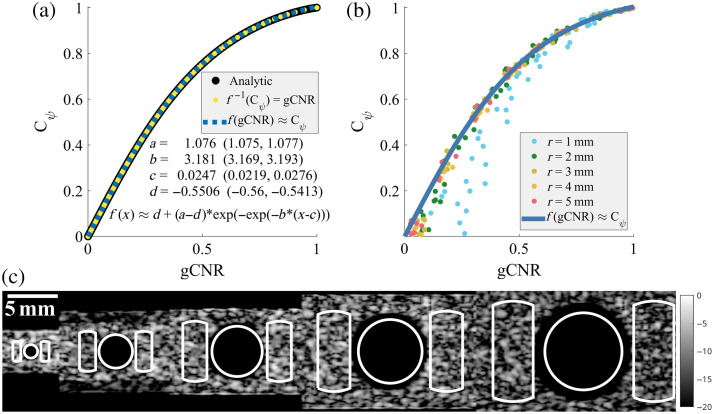
(a) Analytic relationship between gCNR and $C_{\psi }$. The black line is generated from Eqs. (5) and (12) for a variety of CRs, the yellow line is Eq. (13) for $f^{-1}$ for $C_{\psi }$ values between 0 and 1, and the blue line shows the approximation of f included in the figure. (b) Comparing the approximation of f against simulated lesions of varying amplitudes and radii. These simulations show good agreement with the predicted function though for the smallest lesions (r=1  mm), the approximation is weaker as the lesions have higher sampling variability. (c) An example of –30 dB amplitude simulated lesions with radii from 1 to 5 mm. The regions used for image quality metrics are shown in white and are based on 80% of the true lesion radius.

In Sec. 2.3.1, we demonstrated for Rayleigh distributed data how to analytically calculate the relationship of $gCNR=f^{-1}(C_{\psi })$ with Eq. (13). Here, we present a more compact equation for general use that uses a Gompertz fit function

f(gCNR)≈−0.5506+1.627exp(−exp(−3.181(gCNR−0.0247))), (15)

where f was approximated from data generated from the analytic solution in Eq. (13). In Fig. 3, this estimated fit of f is shown with both theoretical values for Rayleigh distributions [panel (a)] and actual simulated ultrasound DAS data [panel (b)]. A Gompertz function was chosen for the fit as it had the best balance of relatively few terms (low complexity) and high accuracy of the estimate (maximum absolute error for any point was 0.00425). Figure 3 also shows this same line plotted over simulated lesions ranging in amplitude from –30 to 0 dB and radii from 1 to 5 mm. The line generally shows good agreement with the simulated DAS data; though as the simulated lesions get smaller, the deviation is noticeable, which makes sense given that the smaller lesions have fewer independent speckle regions (more sampling variability) and more potential sidelobe clutter.

### 4.2 Resolution and Number of Independent Samples

**Fig. 4:**
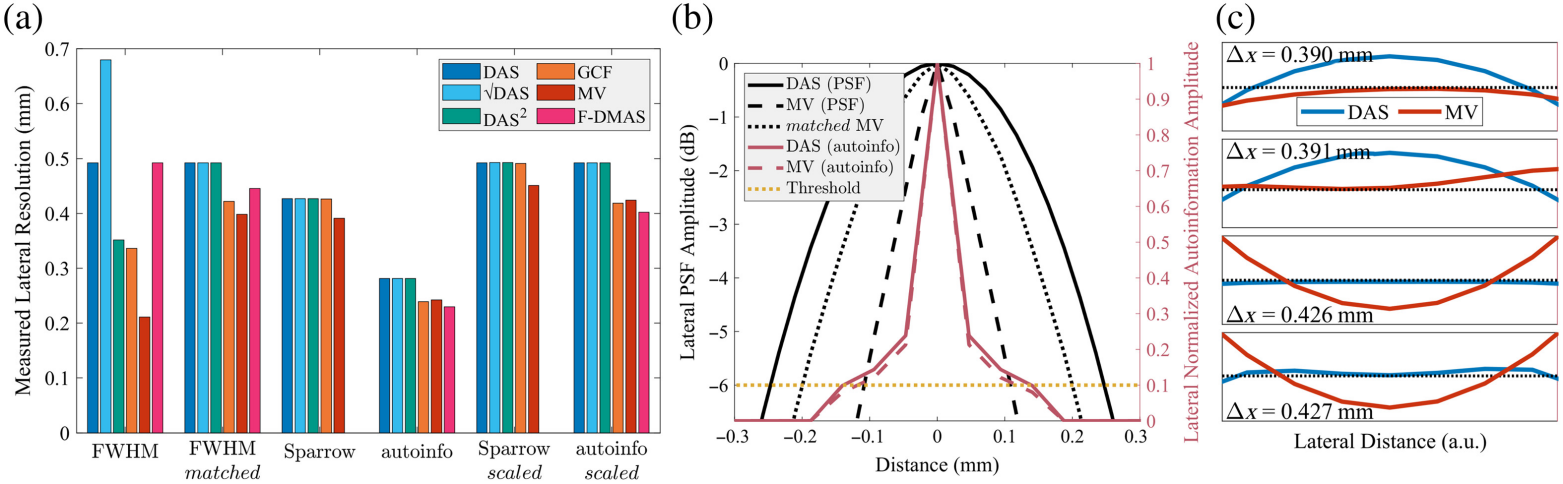
(a) Measured lateral resolution for all beamformers and post-processors using the FWHM of the lateral PSF, FWHM after matching to DAS, Sparrow’s criterion for the resolution limit, and the lateral autoinformation length. In addition, Sparrow’s criterion and the autoinformation length are shown scaled to match the DAS FWHM resolution value. Both Sparrow’s criterion and the autoinformation show similar relative performance between the beamformers, whereas the FWHM shows more variability. Note that F-DMAS has functionally infinite lateral resolution using Sparrow’s criterion as a minimum can always be detected given sufficient lateral sampling. As a result, the Sparrow F-DMAS results cannot be shown. (b) The PSF for DAS, MV, and MV histogram matched to DAS, plotted alongside the autoinformation curves for DAS and MV. The suggested threshold for resolution is marked at the −6  dB line for the PSF and at 0.1 for the normalized autoinformation amplitude. (c) Examples for measuring the lateral resolution with Sparrow’s criterion for DAS and MV. The value of Δx indicates the separation of the point targets, and the separation when a minimum becomes detectable is considered the resolution limit. The axis units are arbitrary as only the presence of a minimum is being measured. A small horizontal dotted black line is included to help show the minimum.

For completeness, M, the number of independent correlation cells in the lesion ROI, and $\overset{˜}{M}$, the robust variant, were calculated for each beamformer using all of the methods described earlier (refer to Table 2). The standard method from Wagner et al.^12^ is to use $M_{\mathrm{FWHM}}$, which uses the FWHM of the lateral PSF and the pulse length. As with $C_{\psi }$, this measurement has been demonstrated to work for DAS data but is unlikely to be an accurate measure for other beamformers. To test this, histogram matching was applied to all cases to match them to the DAS data. These methods are denoted by “FWHM” and “${\mathrm{FWHM}}_{\text{matched}}$,” respectively. Sparrow’s criterion (“Sparrow”) and the autoinformation length (“autoinfo”) were also used as alternative measures. Finally, scaled versions of Sparrow’s criterion (“${\text{Sparrow}}_{\text{scaled}}$”) and the autoinformation length (“${\text{autoinfo}}_{\text{scaled}}$”) were included as well. A summary of all of these methods applied to lateral resolution is included in Fig. 4(a). Figure 4(b) shows a comparison of FWHM, ${\mathrm{FWHM}}_{\text{matched}}$, and the autoinformation length for DAS and MV. Finally, Fig. 4(c) shows an example of evaluating Sparrow’s criterion. F-DMAS unfortunately cannot be effectively measured using Sparrow’s criterion because as long as there was a beam in between the two simulated point targets (which there was by design), a minimum could be detected, making it impossible to measure the “true” resolution. As a result, F-DMAS results that rely on Sparrow’s criterion are not shown in any figures.

**Fig. 5:**
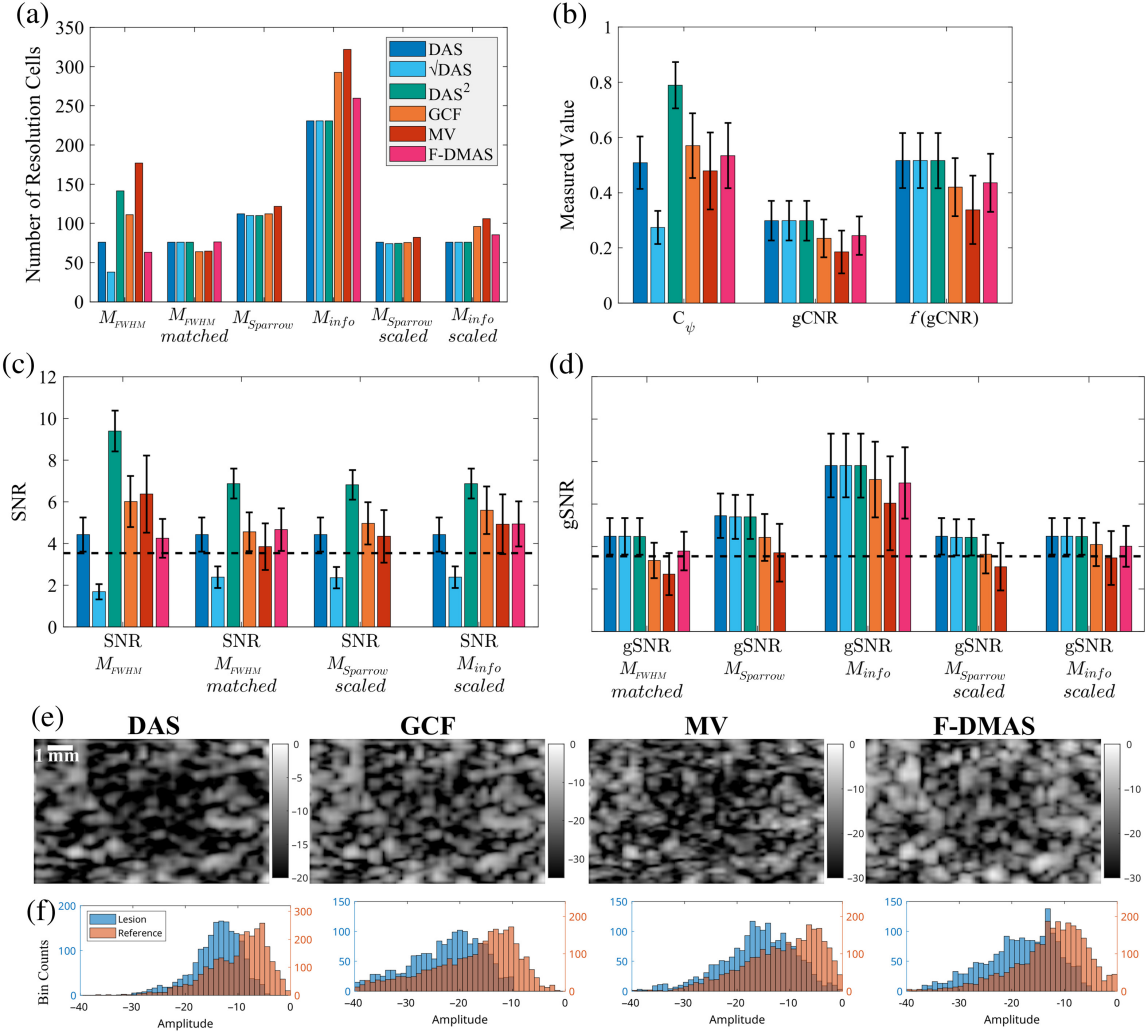
Summary of results for –4 dB lesions, with true radius r=2  mm, ROI radius at 80% of the true radius, and standard deviations shown where applicable. (a) The number of resolution cells was estimated using the FWHM of the PSF, FWHM after matching to DAS, Sparrow’s criterion, the autoinformation length, and both Sparrow’s criterion and the autoinformation length scaled to match the DAS FWHM resolution values. (b) A comparison of $C_{\psi }$, gCNR, and f(gCNR) is shown to demonstrate the effect of using f(gCNR) versus $C_{\psi }$. (c) SNR and (d) gSNR calculated with the different resolution cell estimates as indicated. The dashed line at 3.54 is the Rose criterion reference. As mentioned earlier, F-DMAS results that rely on Sparrow’s criterion cannot be shown. (e) b-mode examples of one realization of the −4  dB r=2  mm lesions that is representative of the average results. (f) The corresponding histogram plots of the lesion and reference areas used to calculate gCNR for the b-mode cases shown. The y-axis is scaled in these plots based on the number of pixels in each area, to correctly show the relative overlap between the two histograms.

Table 3 shows the measured axial resolution for all cases using the same resolution methods. The resolution cell size was then measured as the ellipse formed by the average lateral resolution and the average axial resolution. Figure 5(a) then shows the estimated number of resolution cells in the r=2  mm radius lesions, which is calculated from the size of the lesion (based on 80% of the true lesion radius) divided by the resolution cell size.

**Table 3:** Measured axial resolution (mm).

	DAS	$\sqrt{\mathrm{DAS}}$	${\mathrm{DAS}}^{2}$	GCF	MV	F-DMAS
FWHM	0.385	0.558	0.289	0.385	0.385	0.462
${\mathrm{FWHM}}_{\text{matched}}$	0.385	0.385	0.385	0.533	0.558	0.424
Sparrow’s criterion	0.300	0.306	0.306	0.301	0.302	0.290
Autoinformation length	0.221	0.221	0.221	0.205	0.184	0.241
Sparrow’s criterion “scaled”	0.385	0.393	0.392	0.386	0.388	0.372
Autoinformation length “scaled”	0.385	0.385	0.385	0.357	0.320	0.418

### 4.3 gSNR of Simulated Lesions

As N, the number of independently compounded images, is simply 1 for all cases here, we can now calculate SNR and gSNR for all of our cases in various combinations using Eqs. (1) and (3). Figure 5(a) shows the measured values for a number of resolution cells (M and $\overset{˜}{M}$), and Fig. 5(b) shows $C_{\psi }$, gCNR, and f(gCNR), specifically for the r=2  mm radius and –4 dB amplitude simulated lesions. r=2  mm was chosen as the smallest size that seemed to accurately follow the analytic relationship between $C_{\psi }$ and gCNR (from Fig. 3), and −4  dB was the lowest amplitude that was still somewhat visible in b-mode images and close to the Rose criterion threshold SNR of 3.54. The summary of SNR and gSNR for these cases for each resolution method is shown in Figs. 5(c) and 5(d). In particular, SNR measurements always use $C_{\psi }$, and gSNR always use f(gCNR), but multiple versions of each calculation are shown using different methods for estimating the number of resolution cells, as indicated. B-mode images of one case that followed the average trends are shown in Fig. 5(e) for the distinct beamformers, and the corresponding histograms that reflect the overlap for gCNR are shown in Fig. 5(f).

### 4.4 gSNR of *In Vivo* Liver Case

**Fig. 6:**
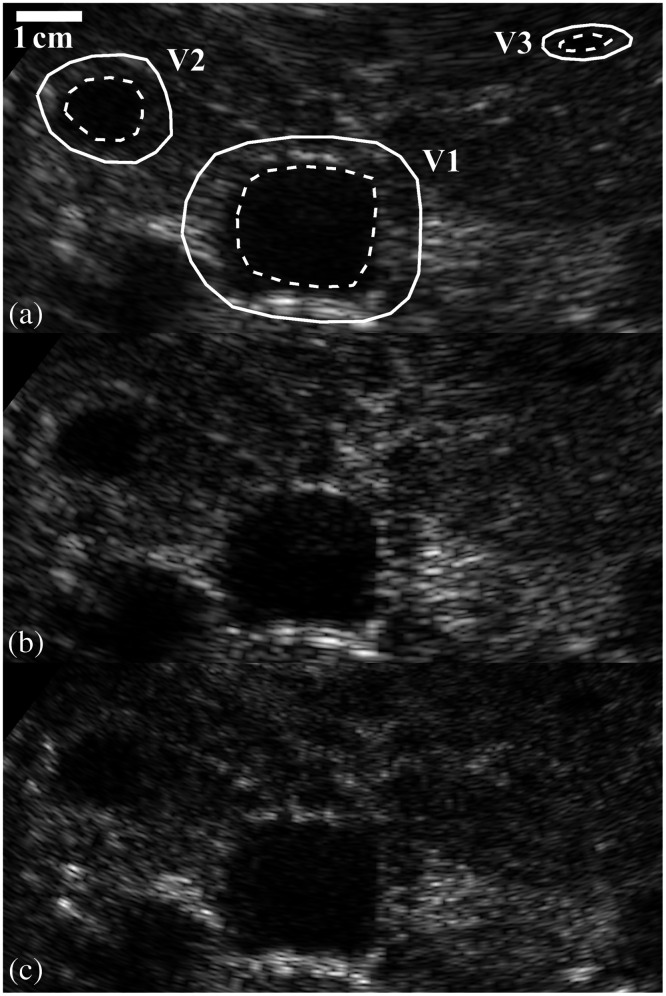
b-Mode images of *in vivo* liver case with vessel ROIs shown with dashed white lines and the corresponding reference backgrounds shown with solid white lines. The same ROI is used for each beamformer case. (a) DAS, (b) GCF, and (c) F-DMAS are included.

An *in vivo* liver case with several differently sized vessels is shown in Fig. 6 for DAS, GCF, and F-DMAS. The specific measurements for SNR and gSNR for each of the three vessels are included in Table 4 for those beamformers along with the transformation ${\mathrm{DAS}}^{2}$. V1, V2, and V3 denote the vessels in descending size. FWHM and Sparrow’s criterion were not used in these cases as each requires specific phantoms to measure, which may not always be available. In comparison, both autocorrelation ($M_{\text{corr}}$) and autoinformation ($M_{\text{info}}$) only require some relatively homogeneous speckle to estimate. For reference, the middle-sized vessel (V2) is around the same size (number of resolution cells) as the r=2  mm–simulated lesion shown in Fig. 5. The Rose criterion threshold of 3.54 still applies to these data.

**Table 4:** *In vivo* measurements of SNR and gSNR.

V1	$C_{\psi }$	$M_{\mathrm{corr}}$	SNR	gCNR	${\overset{˜}{M}}_{\text{auto}}$	gSNR
DAS	0.996	328.9	18.1	0.800	328.9	17.2
${\mathrm{DAS}}^{2}$	1.000	388.1	19.7	0.807	319.8	17.0
GCF	1.000	277.6	16.7	0.723	258.1	14.7
F-DMAS	0.998	511.6	22.6	0.763	460.5	20.0
V2	$C_{\psi }$	$M_{\mathrm{corr}}$	SNR	gCNR	${\overset{˜}{M}}_{\text{auto}}$	gSNR
DAS	0.975	87.8	9.3	0.733	87.8	8.6
${\mathrm{DAS}}^{2}$	1.000	103.6	10.2	0.738	85.4	8.5
GCF	0.996	74.1	8.6	0.723	68.9	7.6
F-DMAS	0.988	136.6	11.7	0.683	123.0	9.9
V3	$C_{\psi }$	$M_{\mathrm{corr}}$	SNR	gCNR	${\overset{˜}{M}}_{\text{auto}}$	gSNR
DAS	0.926	13.7	3.7	0.678	13.7	3.3
${\mathrm{DAS}}^{2}$	0.996	16.1	4.0	0.684	13.3	3.2
GCF	0.986	11.5	3.4	0.680	10.7	2.9
F-DMAS	0.959	21.2	4.6	0.748	19.1	4.0

## 5 Discussion

Perhaps one of the most interesting results of this work is the demonstration that for Rayleigh distributed data, i.e., DAS data, gCNR, and $C_{\psi }$ are functions of one another. In fact, as both metrics are calculated directly from the scale parameters of the Rayleigh distributions, both values are functions of CR as well. This does not diminish the value of these metrics. gCNR was designed with non-Rayleigh distributed data in mind precisely because non-Rayleigh data behave so unpredictably with traditional image quality metrics like $C_{\psi }$. The inclusion of the gray level transform methods demonstrates this clearly in Fig. 5, where arbitrarily squaring the enveloped data results in significant improvements to both $C_{\psi }$ and lateral resolution as measured with the FWHM of the PSF, which in turn increases SNR. As squaring the data does not improve diagnostic information (which we can see by simply re-scaling the dynamic range of these images), these are clearly erroneous results and are a consequence of the non-Rayleigh distributed data. With this in mind, one of the primary criteria for this work was creating a version of SNR that correctly judges these transform methods as equivalent to DAS.

We defined a generalized version of Smith et al.’s SNR,^2^ with Eq. (3), using an analytically solved conversion between $C_{\psi }$ and gCNR using Eqs. (13) and (15). Though the analytic equation is unwieldy to apply, Fig. 3 shows a more simple fitted equation that had a maximum absolute error of 0.00425 from the true theoretical value, which is well within the expected variance of a gCNR estimate from previous work.^14^ The same figure showed this approximation plotted against simulated DAS data, which generally agreed with the expected values. This demonstrates that our analytic solution is correct and that we can substitute $C_{\psi }\approx f(\mathrm{gCNR})$ for an immediate increase in robustness to SNR.

The predicted $C_{\psi }$ for a measured gCNR in Fig. 3 does deviate somewhat for the smallest simulated lesions (r=1  mm) and to a lesser extent lesions where the measured gCNR is low. This is unsurprising for several reasons. First, smaller lesions are more proportionally impacted by off-axis clutter, making them deviate from Rayleigh distributions. Second, smaller sample sizes generally are less likely to accurately represent the population from which they are drawn, which leads to a positive bias compared with the true gCNR value. Finally, low values of gCNR, where the two regions have very similar true distributions, also tend to produce a positive bias, as seen in the figure. As the size of the lesion increases, these effects are diminished, and the estimator converges on the true gCNR. We observed similar things in our previous work, where the smaller lesions frequently produced “incorrect” gCNR estimates compared with the true values used to generate the data.^14^ However, this is an issue for many image metrics, and CNR and $C_{\psi }$ likewise generate “incorrect” estimates for 1 mm lesions compared with the larger lesions that generally are more consistent. We have shown through the results that gSNR is a functional equivalent to SNR for DAS in small and low-contrast lesions, showing that gSNR is working as intended in these scenarios. However, these observations do suggest that both SNR and gSNR may overestimate the likelihood of detection in cases with low contrasts or small sample sizes. While important to keep in mind, the nuances of this bias are outside the scope of this work and likely have a minor impact in most situations.

We demonstrated three different ways of estimating the number of resolution cells in the lesion: $M_{\mathrm{FWHM}}$ using the more common FWHM of the lateral PSF, ${\overset{˜}{M}}_{\text{Sparrow}}$ using Sparrow’s criterion, and ${\tilde{M}}_{\mathrm{info}}$ using the autoinformation length. Sparrow’s criterion is naturally robust against transformations as a local peak in between two points cannot be induced or removed simply via transformations, and likewise, autoinformation is inherently information-based. However, the FWHM, as a measure of autocorrelation, is susceptible to transformations, so to remedy this, we applied histogram matching with DAS as the reference envelope for every case. This effectively removes any transformations in the data and produces a similar relative measure between beamformers similar to the two more robust methods. This is apparent from Fig. 4, where ${\overset{˜}{M}}_{\mathrm{FWHM},\text{matched}}$ is more consistent with the ${\overset{˜}{M}}_{\text{Sparrow}}$ and ${\overset{˜}{M}}_{\text{info}}$ methods. In comparison, the FWHM on the raw data would suggest that several beamformers, including ${\mathrm{DAS}}^{2}$, are significant improvements compared with DAS, and at least in the case of ${\mathrm{DAS}}^{2}$, this is clearly false. The ${\overset{˜}{M}}_{\text{Sparrow}}$ and ${\overset{˜}{M}}_{\text{info}}$ methods produce similar results; however, for DAS, both methods tend to overestimate size than when using $M_{\mathrm{FWHM}}$. As it is assumed that $M_{\mathrm{FWHM}}$ is an accurate measure of the number of resolution cells from the work of Wagner et al.,^12^ a scalar was calculated to scale ${\overset{˜}{M}}_{\text{Sparrow}}$ and ${\overset{˜}{M}}_{\text{info}}$ such that the measured size of DAS was the same as $M_{\mathrm{FWHM}}$. This scaled measurement is likely to be more accurate in the context of measuring the resolution cell size.

A lingering concern is whether resolution cell size can be used as an approximation of correlation cell size in non-Rayleigh situations. Although we use resolution cell size to calculate SNR, technically, it is only a substitution for correlation cell size, and it happens to have been shown that the two are equivalent for Rayleigh distributed data. For the other non-DAS cases here, we have assumed that resolution cell size is more robust compared with correlation cell size for arbitrary distributions. Fig. 4(a) shows that both Sparrow’s criterion and autoinformation length predict similar relative performance between methods, and as autoinformation is similar to a robust autocorrelation measurement,^16^ this might indicate that the resolution cell size here is a better fit for our work. We know that DAS has a directly related resolution cell and correlation cell size, so Sparrow’s criterion should be a good approximation for DAS for correlation cell size. The histogram matched FWHM, scaled Sparrow’s criterion, and scaled autoinformation all measure similar relative performance among the methods. This in turn might imply that they are similarly good approximations of the correlation cell for those other methods. This suggests that a robust resolution cell estimate is likely still a good approximation for use in SNR and gSNR.

The final results in Figs. 5(c) and 5(d) suggest that regardless of SNR or gSNR, larger lesions and higher amplitude lesions will likely be well above the Rose criterion threshold of 3.54 because a radius of 2 mm and an amplitude of –4 dB are already at or above the threshold. The gSNR values calculated with ${\overset{˜}{M}}_{\mathrm{FWHM},\mathrm{matched}}$, ${\overset{˜}{M}}_{\text{Sparrow},\text{scaled}}$, and ${\overset{˜}{M}}_{\text{info},\text{scaled}}$ are consistent and subjectively seem in line with expectations given the b-mode examples in Fig. 5(e). gSNR suggests that GCF and MV actually produce less identifiable lesions than DAS in this case, despite the traditional SNR with $M_{\mathrm{FWHM}}$ showing improvements. Likewise, all three correctly determine that the gray level transform methods are just transformations of DAS. The *in vivo* example in Fig. 6 further confirms these observations and demonstrates that gSNR can translate into *in vivo* applications without issue. In this example, F-DMAS is consistently an improvement compared with DAS due to improved resolution, despite relatively similar $C_{\psi }$ and gCNR measures. Furthermore, all vessels have much higher $C_{\psi }$ and gCNR than the simulated lesions, resulting in consistently higher SNR and gSNR measures at similar sizes.

An important goal was to relate gSNR back to the subjective measurements that SNR was based on, and the fact that gSNR and SNR are the same for DAS is good evidence that it does. Likewise, $C_{\psi }$ and f(gCNR) being nearly identical for DAS are further support. This suggests that for DAS, gSNR can relate back to those subjective assessments the same as SNR, which should be the case as the conversion between the two metrics was mathematically proven. Although the resolution cell assessment of M is generally applicable to other beamformers, the function f to relate $C_{\psi }$ and gCNR was specifically solved for Rayleigh distributions. Yet, it is likely impossible to analytically solve for a similar function for any arbitrary distribution. However, gCNR does provide a non-parametric comparison between any arbitrary distributions and is able to compare relative performance between methods in a robust manner. If an arbitrary beamformer has the same resolution cell size and gCNR as DAS, it seems like a reasonable assumption that both methods would have similar detectability and therefore similar gSNR. From this perspective, using the same f for all beamformers to relate gCNR back to the original SNR and Rose criterion scale makes sense.

It is difficult to be sure of exactly what the Rose criterion threshold for SNR should be, but the generally recommended value of 3.54 does provide a decent guideline for when gSNR measurements begin to reliably indicate the presence of a lesion. However, even without assessing the lesion detection problem, the measurements between beamformers also provide a more accurate sense of the relative performance of adaptive beamformers, compared with using other traditional metrics. gSNR provides a good balance of being non-parametric and therefore robust against transformations while still being linked to earlier works that were designed around subjective clinical assessments, which are critical for researchers and industry professionals. This suggests that gSNR should correlate with overall image quality while also being more broadly applicable to adaptive beamformers.

From the data presented, gSNR likely does have limitations beyond which it (and SNR) will struggle. Specifically, sampling theory indicates that these methods will be less accurate and overall more variable for smaller lesions, and there is no obvious solution to correct this. This high variance is a greater challenge for targets at low contrasts due to the increasingly asymmetrical nature of the distribution of the metric as contrast lowers. Separately, all of the metrics considered here operate on the enveloped data and therefore can only predict the quality of the enveloped data. Enveloping data results in a loss of information, and image quality metrics designed to work with the pre-enveloped data would provide a deeper look into beamformer performance. However, given the heavy assumptions in the derivations of both SNR and gSNR on the nature of the enveloped data, it is difficult to predict how or even if gSNR could be translated to work in that domain.

## 6 Conclusions

In this work, gSNR, a robust version of Smith et al.’s SNR,^2^ is proposed. We demonstrate that it is possible to find an analytic relationship between $C_{\psi }$ and gCNR for Rayleigh distributed data, from which we find an empirical estimate of the function $f(\mathrm{gCNR})=C_{\psi }$, allowing the use of gCNR instead of $C_{\psi }$. We additionally show multiple methods for calculating the resolution cell area in a more robust manner. Using gCNR and a robust resolution cell estimate to calculate gSNR provides a more consistent estimate of lesion detectability across beamformers. The gSNR metric applied to DAS data shows similar results to SNR as intended but has the benefit of being more robust for other adaptive beamformers such as GCF, MV, and F-DMAS, as shown here. gSNR is demonstrated to work with *in vivo* data as well without any additional considerations, as long as the resolution can be robustly measured.

## Data Availability

The data were generated via simulations with the parameters included. Data are available from the authors upon request. The code used to calculate gCNR is available in the GitHub repository listed in Sec. 2.2: https://github.com/VU-BEAM-Lab/gCNR_solver.
